# Impact of High-Altitude Hypoxia on Bone Defect Repair: A Review of Molecular Mechanisms and Therapeutic Implications

**DOI:** 10.3389/fmed.2022.842800

**Published:** 2022-05-10

**Authors:** Pei Chen, Yushan Liu, Wenjing Liu, Yarong Wang, Ziyi Liu, Mingdeng Rong

**Affiliations:** ^1^Department of Periodontology and Implantology, Stomatological Hospital, Southern Medical University, Guangzhou, China; ^2^Department of Prosthodontics, Stomatological Hospital, Southern Medical University, Guangzhou, China

**Keywords:** hypoxia, plateau, bone defect, bone regeneration, high altitude

## Abstract

Reaching areas at altitudes over 2,500–3,000 m above sea level has become increasingly common due to commerce, military deployment, tourism, and entertainment. The high-altitude environment exerts systemic effects on humans that represent a series of compensatory reactions and affects the activity of bone cells. Cellular structures closely related to oxygen-sensing produce corresponding functional changes, resulting in decreased tissue vascularization, declined repair ability of bone defects, and longer healing time. This review focuses on the impact of high-altitude hypoxia on bone defect repair and discusses the possible mechanisms related to ion channels, reactive oxygen species production, mitochondrial function, autophagy, and epigenetics. Based on the key pathogenic mechanisms, potential therapeutic strategies have also been suggested. This review contributes novel insights into the mechanisms of abnormal bone defect repair in hypoxic environments, along with therapeutic applications. We aim to provide a foundation for future targeted, personalized, and precise bone regeneration therapies according to the adaptation of patients to high altitudes.

## Introduction

Due to commercial activities, military deployment, tourism, and entertainment, high-altitude areas (altitude ≥2,500–3,000 m) are among the most important residential and business spaces for modern humans. There are three major plateaus in the world, including the Tibetan Plateau, the Andes Mountains, and the Ethiopian Plateau. The total plateau area is estimated at more than 11,000,000 km^2^, with a population of ~107 million. The main characteristics of the high-altitude environment include (1) low pressure, oxygen deficiency, and thin air; (2) cold, dry, and strong winds; and (3) long sunshine time and intense ultraviolet radiation. Specifically, the main factors affecting the body are thin air, low atmospheric pressure, and reduced oxygen partial pressure in a high-altitude environment.

There is a relationship between altitude and the decreased partial pressure of oxygen (PO_2_; i.e., the tension produced by oxygen dissolved in the blood). At an altitude of ~3,000 m, although arterial PO_2_ (PaO_2_) is reduced, oxygen saturation can be well maintained (1). The high-altitude environment affects humans mostly because reduced PaO_2_ in the blood leads to hypoxia (2, 3). At sea level plains (henceforth referred to simply as “plains”), the PaO_2_ is ~21.15 kPa. However, as the altitude increases by 100 m, atmospheric pressure decreases by 5.9 mmHg, and PaO_2_ decreases by 1.2 mmHg (4). When the pressure in the air inhaled by humans is lower than 16 kPa (2,500–3,000 m), the symptoms of hypoxia start to appear, including increased breathing and heart rate, headache, loss of appetite, poor sleep, decreased exercise ability, and even mountain sickness (5).

The major metabolic phases of bone defect healing overlap with biological stages, including several stages: haematoma, unmineralized cartilage (soft callus), fibrous tissue, sencodary bone (hard callus), remodeled bone (6). Bone defect repair is affected in high-altitude environments. Indeed, the high-altitude environment has a systemic effect on humans, negatively impacting bone mass, microstructure, and biomechanics of normal bone (7), resulting in declined repair ability of bone defects as follows. First, the healing time of bone defects is significantly prolonged. The incidence of non-union at plateaus is 20–30%, which is significantly higher than that of plains (0.4%) (8). Bone repair efficiency is also significantly reduced (9), and fracture healing time is significantly longer, especially at extreme altitudes (5,400–6,700 m), than that found in coastal areas (10). Furthermore, X-ray findings reveal no periosteal hyperplasia and bony callus generation at the fracture end, while osseointegration is also poor after implantation (9, 11). Second, the different physiological components of bone defect healing are compromised. The capacity to regenerate bone is weaker based on the structural, geometrical, and material properties in a high-altitude environment (12). In addition, callus reconstruction is difficult, and the bone defect is filled with less callus tissue, with mostly new bone and cartilage (13). Moreover, the number of osteoblasts is reduced, and bone defect healing mainly involves endochondral bone (14). Third, different challenges are faced as part of bone defect treatment. For instance, in high-altitude areas, due to the special natural geographical environment of plateaus, the treatment of bone defects caused by war trauma differs from therapies applied in low altitude areas (15). After autogenous bone transplantation, hyperbaric oxygen therapy (HBOT) is often needed to achieve a relatively high success rate (16).

Herein, we review the physiological factors influencing bone repair, the cellular mechanisms of abnormal bone repair, and therapeutic research progress made in addressing the impact of a high-altitude environment on bone defect repair.

## Factors Influencing Bone Defect Repair in High-Altitude Environments

**Figure 1:**
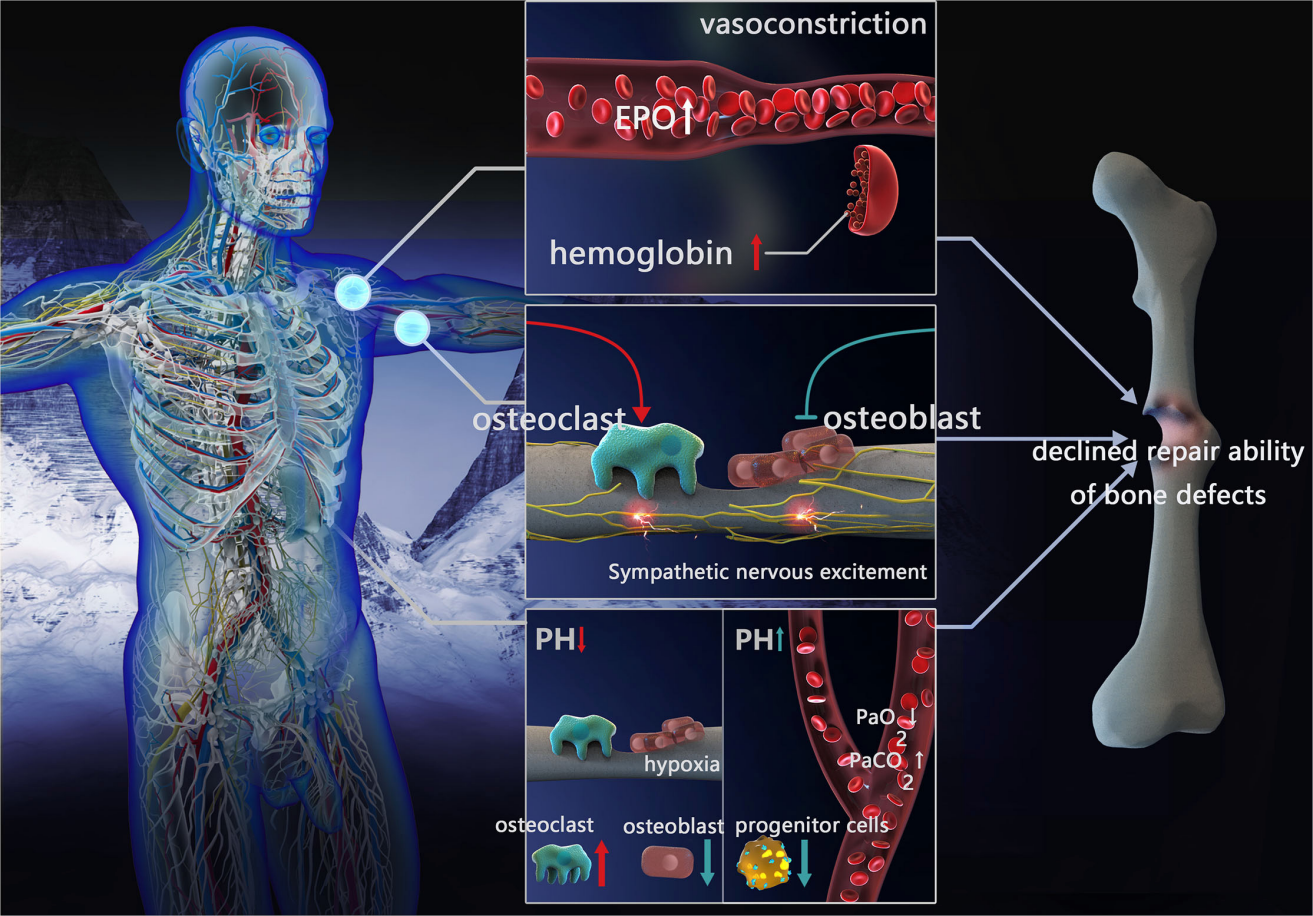
Factors influencing repair of bone defects in high-altitude environment. Blood compensation: vessels constrict, EPO concentration and hemoglobin increased. Sympathetic nervous excitement: inhibit osteoblast activity and promote osteoclast formation. Acid-base compensation: In local acid-base compensation, decreased pH impeding osteoblasts differentiation and enhanced osteoclast activation. In systemic acid-base compensation, with the decreasing of PaO_2_ and increasing of PaCO_2_, increased pH inhibits bone marrow progenitor cell proliferation (EPO, erythropoietin; PaCO_2_, partial pressure of arterial CO_2_; PaO_2_, partial pressure of oxygen).

The high-altitude environment can exert systemic effects on humans that presents as a series of compensatory reactions. Although these compensations are conducive to adaptation to low pressure and oxygen at high altitudes, they may affect bone defect repair (Figure 1).

### Blood Compensation

The blood system is among the first responses to the high-altitude environment, leading to a series of compensatory reactions. When staying at a high altitude for a short time, the pulmonary arterioles contract, pulmonary blood flow resistance increases, pulmonary arterial pressure increases, and volume vessels contract (peripheral veins), resulting in a release of reserved blood, thus ensuring blood supply to important organs (heart and brain). After living on a plateau for a longer time, acclimatization to the environment occurs by increasing oxygen transport, erythropoietin (EPO) concentration, the number of red blood cells, and hemoglobin concentration (17). However, when the physiological capacity to adapt is exceeded, maladaptation occurs *via* increased blood viscosity, reduced oxygen transport and oxygen release to vital tissues, and further aggravation of tissue hypoxia. Due to genetic differences, the Andes and Han populations living on the elevated altitudes of Tibet are more susceptible to this type of maladaptation, while the Tibetan residents have higher resting and hypoxic ventilation responses (HVRs), lower arterial oxygen saturation, and reduced hemoglobin concentration at the same altitude (18).

Blood compensation may affect the healing of bone defects through increased EPO production in response to the low pressure and oxygen environment at high altitudes. This in turn affects mobilization and differentiation (osteoblasts, osteoclasts, and mature blood cells) in mesenchymal stem cells (MSCs) and hematopoietic stem and progenitor cells (HSPCs). It is known that low EPO concentration is conducive to bone formation and bone defect healing (19, 20), while high EPO levels lead to the stimulation of osteoclast precursors and induces bone loss, preventing healing (21).

Blood compensation may lead to insufficient blood supply through increased blood viscosity, thus affecting the healing of bone defects. During bone defect healing, the blood vessels and interstitial tissue around the bone need to continuously grow into the center of the defect; hence, the abundance of blood supply affects the healing process. At high altitudes, blood compensation leads to an increase in blood cells. When blood viscosity exceeds a certain limit, it leads to local microcirculation disturbance (22). Platelets in stagnant blood promote the release of thrombin and damage endothelial cells, resulting in increased 5-hydroxytryptamine and histamine content (23), basement membrane exposure, the release of coagulation factors, and even thrombosis (24–26). Therefore, poor blood supply induced by blood viscosity may be one of the factors influencing poor bone defect healing.

### Sympathetic Nervous Excitement

In the high-altitude environment, low oxygen stimulates the sympathetic nervous system, prompts catecholamines secretion, accelerates the heartbeat, enhances myocardial contractility, increases cardiac output, and elevates arterial blood pressure to a certain extent. On the skeleton, the sympathetic nerve fibers run along the major artery and nourish the bones through nutrient pores. Both the periosteum and bone marrow receive nutrients from noradrenergic fibers (often associated with the vascular system), vesicular acetylcholine transporter (VAChT), and vasoactive intestinal polypeptide immunoreactive fibers (often associated with the parenchyma). Sympathetic fibers on the periosteum branch overlaying the bone marrow and dense mineralized bone region receive the greatest mechanical stress and load, while also having the highest metabolic rate. Furthermore, the periosteum is the site with the most abundant blood vessels and has the highest density of sympathetic and sensory fibers.

Sympathetic nervous excitement may affect bone defect repair through vasoconstriction, leading to an inadequate blood supply. In high-altitude environments, sympathetic nerves are excited, form a large number of sympathetic active substances, and act on the vascular smooth muscle α receptor, leading to vasoconstriction, enhanced resistance of surrounding blood vessels, and reduced perfusion flow of the tissue (27). Furthermore, it results in decreased blood supply and hypoxia in the soft tissue at and around the bone defects, affecting hematoma organization and callus formation, and interrupting the healing of the bone fracture. The effect of vasoconstriction on the reduction of local blood flow in bone defects is more pronounced with increasing altitude (28).

Sympathetic nervous excitement regulates bone homeostasis and promotes bone resorption. Sympathetic nervous excitement at high altitude may result in noradrenaline nerve endings releasing noradrenaline and stimulating β2-adrenergic receptor (β2AR) near osteoblasts and osteocytes, bone formation inhibition, increased receptor activator of nuclear factor-κB ligand (RANKL) expression, promotion of osteoclast formation, and increased bone resorption. Altogether, these effects impede bone formation. Lastly, the secretion of adrenergic agonists (catecholamines) also stimulates bone resorption, inducing bone loss (29).

### Acid-Base Compensation

High-altitude hypoxia induces hyperventilation and increases pH, due to the decreased oxygen availability and subsequent lower PaO_2_. The HVR occurs when the body attempts to maintain PaO_2_ to adapt to the high altitude. Although the PaO_2_ level during HVR is corrected to a certain degree, partial pressure of arterial CO_2_ (PaCO_2_) is simultaneously reduced, further leading to decreased plasma H_2_CO_3_ concentration and increased pH (30). Minor changes in pH have negligible physiological effects, however, when the pH keeps increasing, a compensatory acid retention mechanism and increased excretion of HCO_3_ in urine is triggered, causing diuresis of sodium bicarbonate and potassium bicarbonate (31), in turn leading to decreased pH in arterial blood (pHa) compared to the normal level (pHa ≈ 7.4) (32, 33). Insufficient renal compensation may cause a continuous increase of blood pH, weaken HVR, and reduce oxygen saturation leading to the onset of acute mountain disease.

Nevertheless, local pH change in bone defects differs from that of systemic responses. In a high-altitude environment, due to decreased vascular perfusion, tissue hypoxia generates an acidic environment in the bone defect area. Moreover, when a bone defect occurs, disruption of the blood supply can have negative consequences for the bone *via* the direct actions of hypoxia and acidosis on bone cells (34). In severe hypoxia, glycometabolism at the injury site is incomplete, anaerobic glycolysis is enhanced, and local acidic products are increased (35).

Healing of the bone defect is affected by systemic and local acid-base compensation. A continuous increase of physiological pH caused by the high-altitude environment affects oxygen-carrying capacity, as well as the transportation and release of hemoglobin and blood. In addition, it increases the affinity of hemoglobin to oxygen (Bohr effect) and allows tissues to absorb more oxygen (33). Therefore, in terms of oxygen delivery to the bone tissue, the acid-base compensatory response under the high-altitude environment is advantageous for increasing the local oxygen content in bone defects. However, the pH increase resulting from this compensation does not promote bone marrow progenitor cell proliferation and may affect bone defect healing. Studies have reported that promyelocytic KG-1a cells (hematopoietic stem cells) cultured under high pH have significantly decreased proliferation and enhanced apoptosis (35). The bone contains a large number of alkaline minerals (hydroxyapatite), and bone cells are extremely sensitive to the direct effects of pH. When the acid-base balance cannot be maintained within a narrow range, these alkaline minerals can eventually be used to neutralize pH, in turn reducing alkaline mineral deposition by osteoblasts in the bone (36), and impeding osteoblasts differentiation (34, 37). In contrast, the osteoclastic differentiation and activation are enhanced (37, 38) resulting in detrimental bone defect repair.

## Mechanism of Abnormal Bone Defect Repair in High-Altitude

**Figure 2:**
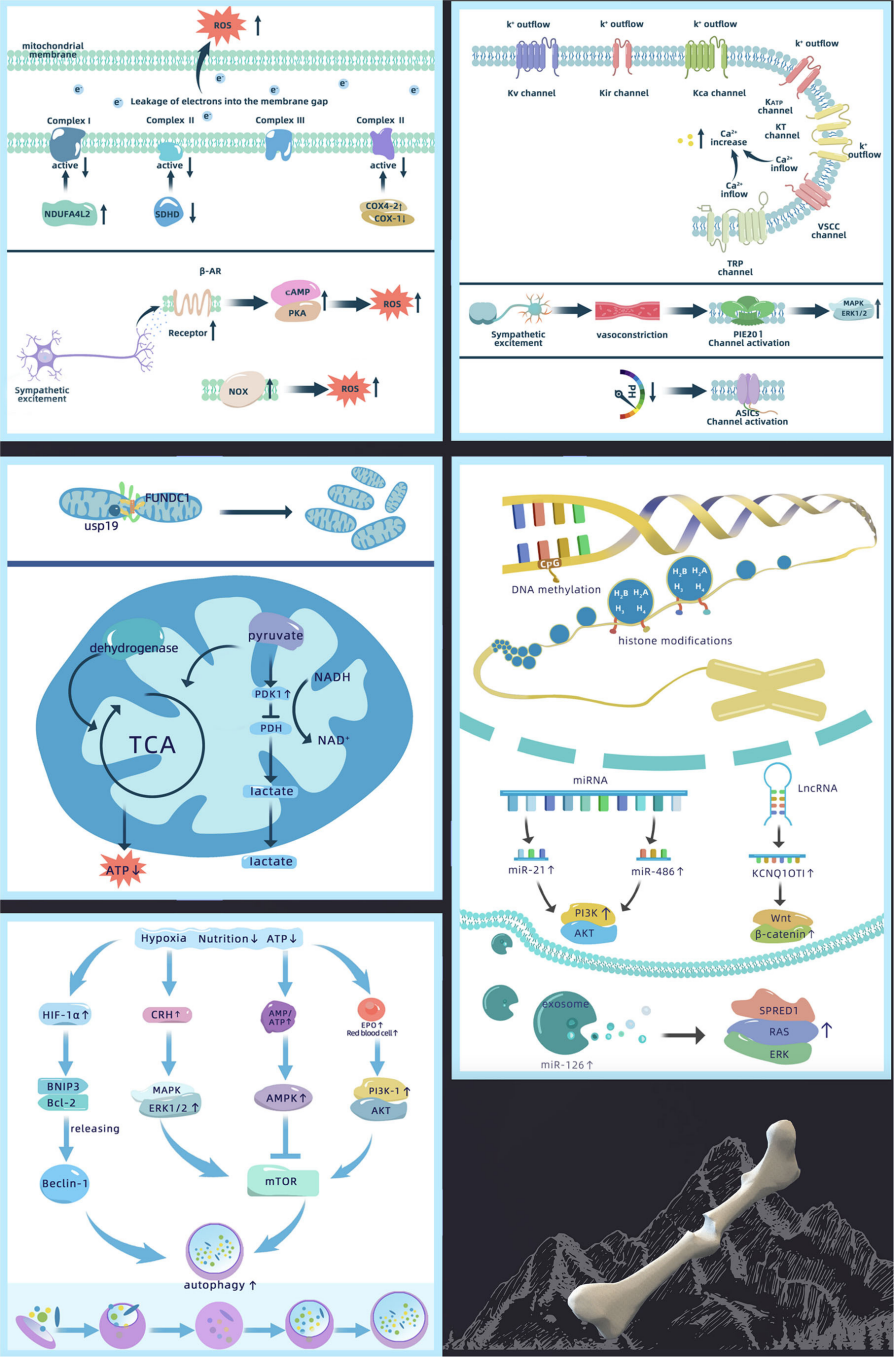
Mechanism of abnormal bone defect repair in high-altitude, including changes in ion permeability of the cell membrane, ROS production, changes in mitochondrial function, activation of autophagy, and epigenetics regulation [Akt, protein kinase B; AMPK, adenosine 5′-monophosphate-activated protein kinase; ASIC, acid sensitive ion channel; ATP, adenosine triphosphate; Bcl-2, B-cell lymphoma 2; BNIP3, Bcl-2 19 kDa interacting protein 3; COX, cytochrome C; CRH, corticotropin releasing hormone; EPO, erythropoietin; ERK1/2, extracellular regulated protein kinases 1/2; FUNDC1, FUN14 domain-containing 1; HIF-1α, hypoxia inducible factor-1α; KATP, ATP-sensitive K^**+**^ channels; KCa, Ca^**2+**^ activated K^**+**^ channel; KCNQ1OT1, potassium voltage-gated channel subfamily Q member 1 opposite strand 1; Kir, inward rectifying K^**+**^ channel; KT, double-pore K^**+**^ channels; Kv, voltage-gated potassium channel; lncRNA, long non-coding RNA; MAPK, mitogen-activated protein kinase; miRNA, micro-RNA; mTOR, mammalian target of rapamycin; NADH, nicotinamide adenine dinucleotide; NDUFA4L2, NADH dehydrogenase (ubiquinone) 1 alpha subcomplex 4-like 2; NOX, NADPH oxidase; PDH, pyruvate dehydrogenase; PDK1, pyruvate dehydrogenase kinase 1; PI3K, phosphoinositide 3-kinase; PKA, protein kinase A; ROS, reactive oxygen species; SDHD, succinate dehydrogenase complex subunit D; SPRED1, sprouty-related EVH1 domain containing 1; TCA, tricarboxylic acid cycle; TRP, transient receptor potential channel; USP19, ubiquitin specific protease 19; VSCC, voltage sensitive Ca^2**+**^ channel].

High-altitude areas have thin air and reduced PaO_2_. Therefore, cellular structures closely related to oxygen sensing, including the mitochondria and cell membrane ion channels, produce corresponding functional changes causing oxidative stress response, ion permeability changes, signaling pathway activity changes, autophagy, apoptosis, etc. In addition, the high-altitude environment alters the epigenetic modification-related effects on genes, thus affecting protein function that may have consequences for bone defect repair (Figure 2).

### Changes in Ion Permeability of the Cell Membrane

Ion channels participate in a variety of high-altitude adaptive compensations such as HVR and vascular tension changes. It is generally accepted that high-altitude environments inhibit K^+^ channels and activate Ca^2+^ channels (39, 40). Altered K^+^ permeability affects osteoblast differentiation and proliferation (41). Meanwhile, altered Ca^2+^ permeability affects the mineralization of extracellular inorganic Ca^2+^, which acts as a second messenger, impacting the expression of downstream osteogenesis-related signaling pathways (42), thereby influencing bone defect repair.

High-altitude-related low oxygen inhibits the activity of K^+^ channels, leading to membrane depolarization. Acute hypoxia depolarizes the membrane potential by 15–20 mV (43). It has been confirmed that K^+^ channels affected by oxygen in bone cells include voltage-gated potassium channels (Kv), inward rectifying K^+^ channels (Kir), Ca^2+^-activated K^+^ channels (K_Ca_), ATP-sensitive K^+^ channels (K_ATP_), and two-pore K^+^ channels (KT) (44). However, the mechanism by which these K^+^ channels sense oxygen changes is not fully elucidated. Studies have found that K^+^ channels retain their hypoxic reactivity after recombination (45, 46), indicating that oxygen not only directly regulates K+ channels but also affects ion permeability through pore-forming subunits or regulatory β-subunits. Six transmembrane subunits and one pore-forming subunit (Kv1.2, Kv1.5, Kv2.1, Kv3.1, Kv3.3, Kv4.2, and Kv9.3) of the Kv channels are reversibly blocked by hypoxia (47). Furthermore, four transmembrane and two pore-forming subunits of the double-pore potassium ion channel KT are also involved in oxygen sensing peripheral chemoreceptors (48). However, whether other subunits play a role in the low-oxygen environment at high altitudes remains unclear.

High-altitude hypoxia increases intracellular Ca^2+^ levels and causes calcium overload, which results from Ca^2+^ channel activation. The main Ca^2+^ channels affected by oxygen in bone cells include voltage-sensitive Ca^2+^ channels (VSCCs) and transient receptor potential channels (TRPs). In these Ca^2+^ channels, TRPs are the most predominant oxygen sensors (49). TRP expression differs on the surface of different bone cells; e.g., TRP vanilloid-5 (TRPV5) is missing in osteoblasts (50). However, only the hypoxic responses of TRPV1 and TRPV4 have been examined. Nevertheless, TRPV5 and TRPV6 are also closely associated with bone defect healing (51). Different from the other TRPV channels, these are highly selective for Ca^2+^ and their changes under hypoxia need to be confirmed. In addition, although VSCC expression is greatly reduced on osteoblasts compared with excitable cells, reduced PaO_2_ can lead to rapid activation of L-type VSCCs (52). However, the change in T-type VSCC under hypoxic conditions has not been reported. This may be a result of the T-type VSCC only being involved at the early stage of cell differentiation, after which its activity significantly reduces (53).

In addition to oxygen-sensitive ion channels, only a few studies have examined changes in other ion channels that may be associated with high-altitude environments. For example, compensatory responses to high-altitude have been reported whereby sympathetic excitation can lead to local vasoconstriction with increased expression of the mechanically-gated channel Piezo1 during vasoconstriction (54), activating the downstream MAPK/ERK1/2 signaling pathways. In addition, the local extracellular environment of bone defects is acidified under hypoxia, which can activate acid-sensitive ion channels (34).

It may be speculated from existing studies that oxygen-sensitive ion channels play the main role in high-altitude bone defects. Most literature assessing the effects of high altitude on ion channels focused on pulmonary edema. However, whether other subtypes of TRPs (such as TRPC, TRPA, TRPM) also present similar changes in bone cells as pulmonary vascular cells have not been reported and should be considered for future research. In addition, there are few studies on Na^+^ channels. The latest study showed that Na^+^ acts as a second messenger to regulate the permeability of the inner mitochondrial membrane in an acute hypoxic environment (55). Therefore, attention should be paid to the changes of Na^+^ channels in high-altitude environments as well as the related effects on bone defect repair.

### ROS Production

Both acute and long-term exposures to high-altitude environments induce the production of reactive oxygen species (ROS), which results in oxidative stress response (56, 57). The ROS level increase in the bone can affect bone defect healing by decreasing osteoblast activity and accelerating bone resorption by osteoclasts (58). In addition, ROS can also induce osteoblast and osteocyte apoptosis (59), affecting the quality of bone defect healing.

Hypoxia is the main factor increasing ROS in high-altitude environments. At a plateau, PaO_2_ gradually decreases with altitude elevation. The mitochondria, a main source of ROS, are affected by the changes in intracellular and extracellular PaO_2_. Multiple studies have suggested that mitochondrial complex III (ubiquinol-cytochrome c oxidoreductase) and complex I (NADH-ubiquinone oxidoreductase) are major sites of ROS production (60–62). Hypoxia reduces complex I activity by upregulating NDUFA4L2 (63). However, studies have found that complex IV (cytochrome C oxidase) and complex II (succinate dehydrogenase) in the mitochondria are also sensitive to hypoxia. This is because complex IV has a binding site for oxygen, and hypoxia can reduce its activity by upregulating COX4-2, an isoform of cytochrome C oxidase (COX), and downregulating COX-1 (64). It also affects the activity of complex II by decreasing SDHD expression (62). In addition, insufficient oxygen supply can also obstruct mitochondrial electron transport by partially leaking single electrons from complexes I–III directly to oxygen leading to the production of large amounts of ROS (56).

In addition to the effects of hypoxia, sympathetic nervous system excitement at high altitude may be one of the factors that contribute to increased ROS production. It was found that adrenaline receptors mediate the production of cellular ROS induced by sympathetic system over-excitation. β-AR promotes the mitochondrial tricarboxylic acid cycle, enhances oxidative respiration, and increases oxygen consumption through the classical cAMP/PKA pathway, which leads to enhanced electron leakage into the mitochondrial inner and outer membrane spaces, thereby increasing ROS production (65). In addition to the mitochondria, NOX (NADPH oxidase), distributed on the plasma membrane and multiple organelle membranes, also produces ROS upon sympathetic system excitation (66) and promotes vasoconstriction through a RhoA kinase-dependent pathway (67). However, studies examining sympathetic nervous excitement with ROS are currently limited to cardiomyocytes and vascular smooth muscle cells. Thus, whether a similar mechanism also exists in bone cells needs confirmation.

The results of *in vitro* mechanistic studies evaluating high-altitude-related ROS production as well as the actual *in vivo* situation in high-altitude populations may differ due to the genetic polymorphisms of ROS production in high-altitude environments. Studies have found that the mtDNA 10609T in Han people living on the plateau promotes the increase of intracellular ROS in hypoxia, while the mtDNA T8414 does not (68). In addition, the high-altitude environment increases ROS, which has a negative effect on bone defects. On the other hand, increased ROS can stabilize the activity of hypoxia-inducible factor 1-alpha (HIF-1α), which is helpful for the body to cope with the high-altitude environment. Therefore, balancing the advantages and disadvantages ROS still needs quantitative investigation.

### Changes in Mitochondrial Function

Mitochondrial dysfunction can affect osteoblast, osteocyte, and vascular endothelial cell functions (69, 70) as well as inflammatory reactions in chondrocytes. In turn, this leads to metabolic disorders in chondrocytes, affecting endochondral osteogenesis (71). Moreover, osteogenic differentiation of human marrow MSCs can be impeded by mitochondrial dysfunction (72), which is not advantageous for bone defect repair.

Mitochondria are the main consumers of oxygen in cells and need to adapt collectively to the decrease in available oxygen at high altitudes. Low oxygen environments can lead to an increased number of mitochondria and imbalanced proteostasis. A possible mechanism is that hypoxia induces mitochondrial fission *via* mitochondrial outer-membrane protein FUNDC1 signaling. Under hypoxia, the deubiquitinase USP19 accumulates at the ER-mitochondria contact sites with FUNDC1. USP19 then interacts with and removes ubiquitin chains from FUNDC1 at the ER-mitochondria contact sites. After USP19 stabilizes FUNDC1 and subsequently promotes Drp1 oligomerization (73, 74), hypoxia-induced mitochondrial division occurs thus increasing the number of mitochondria. In addition, recent studies have found that hypoxia suppresses mTORC1 signaling and mediates homeostasis remodeling of mitochondrial proteins by regulating substrate-related mitochondrial metabolism through the mTORC1-LIPIN1-YME1L signaling axis (75).

In addition, high-altitude environments may also cause changes in mitochondrial respiration and aerobic capacity. However, how this dysfunction relates to the degree of hypoxia is unknown. Mitochondrial respiratory function is not affected in mild or early hypoxia. It was found that the effect of 15 days of mild normobaric hypoxia on mitochondrial function is negligible as mitochondria adapt to the environment by increasing LON protease content, optimizing respiratory chain function (76). However, severe hypoxia leads to mitochondrial dysfunction. When the PaO_2_ in the mitochondria drops to the critical point of 0.1 kPa (<1 mmHg), dehydrogenase activity decreases, reducing the respiratory function of the mitochondria and decreasing ATP production (77). Moreover, mitochondrial metabolic pathways switch from aerobic to anaerobic metabolism under hypoxic conditions. The possible explanation is that hypoxia upregulates pyruvate dehydrogenase kinase 1 (PDK1), inactivates pyruvate dehydrogenase (PDH), transforms pyruvate to acetyl-CoA, and reduces the availability of substrates for oxidative metabolism, thereby promoting the conversion of pyruvate to lactate (78).

However, changes in mitochondrial function under high-altitude environments are correlated with mitochondrial genes. Studies have found that mitochondrial haplogroups B and M7 may be related to inadaptability to hypoxia, while haplogroups G and M9a1a1c1b are related to hypoxia adaptation. Specifically the T3394C and G7697A mutations in haplogroup M9a1a1c1b may be the main factor improving the ability to adapt to the environment in Tibetans living on the plateau for generations (79). In addition, the mitochondrial genes *MT-ND1* and *MT-ND2*, encoding two subunits of mitochondrial NADH dehydrogenase, play an important role in the oxidative phosphorylation electron transport chain and contribute significantly to high-altitude hypoxia adaptation of the mitochondria (80).

In addition to hypoxia, high-altitude factors such as cold (81) and strong ultraviolet radiation (82, 83) may also impact mitochondrial function. Nonetheless, no relevant studies have assessed their role on mitochondria in bone tissue, which may be an important future direction.

### Autophagy

Both acute and chronic high-altitude exposures activate autophagy and increase cell death (84). Overall, autophagy exerts a protective effect under short-term moderate stimulation induced by the high-altitude environment (85, 86), which can increase the expression of vascular endothelial growth factor (VEGF) by stabilizing HIF-1α, thereby benefiting angiogenesis (87). Meanwhile, as a carrier of osteoblasts to secrete hydroxyapatite crystals, autophagosomes participate in bone formation (86, 88). However, a sustained high level of the oxidative stress response can overstimulate autophagy, leading to premature cell death (89).

Studies have found that autophagy acts in a HIF-1α-dependent manner under hypoxia (90). HIF-1α activates downstream BNIF3, then competitively binds Bcl-2 with Beclin-1 for subsequent activation of autophagy. However, the regulatory mechanism involved in autophagy has not been studied in bone cells. Findings in other cell types reveal that hypoxia affects autophagy *via* the HIF-1α/Beclin-1 pathway in SH-SY5Y cells (neuroblastoma cells), dendritic cells (DCs) (91), and vascular endothelial cells (87). However, this autophagy activating pathway is not present in all cell lines. For example, autophagy of NP cells (nucleus pulposus cells) in the hypoxic environment is independent of the HIF-1α pathway. Therefore, further investigation is needed to determine whether the same mechanism exists in skeletal cells.

In addition, a high-altitude environment can also regulate autophagy through the mTOR-related signaling pathway as mTOR kinase can be inhibited under conditions such as malnutrition, decreased ATP levels, and hypoxia. Thus, reduced metabolic activity induces autophagy (90). Cellular energy deficiency resulting from high altitude leads to increased AMP/ATP ratio (92), whereas hypoxia induces the phosphorylation of AMPK (93), all of which regulate the AMPK/mTOR signaling pathway. Moreover, the increased number of red blood cells and EPO caused by high-altitude compensation can activate the PI3K-1/Akt/mTOR signaling pathway (94). The low-oxygen environment at high altitude also upregulates corticotropin-releasing hormone (CRH) and regulates mTOR in autophagy through the MAPK/ERK1/2 pathway (95, 96).

However, the basal level of autophagy is not the same in different populations; hence, the effects of autophagy on bone cells may also differ between individuals in high-altitude environments. Studies have shown significant differences in the levels of autophagy markers, including LC3 and BNIP3, between Tibetans living at an altitude of 3,000 m and Han nationals living below 500 m (97). Long-term living in high-altitude environments could increase the level of basal autophagy—possibly by resisting the negative effects of oxidative stress through a higher level of autophagy.

Besides, bone marrow is in a hypoxic environment under physiological conditions. Thus, whether *in vitro* effects of hypoxia on autophagy signaling pathways could be translated *in vivo* need to be confirmed. However, through literature review, we found that altitude affects autophagy by various mechanisms, emphasizing the complexity of the autophagy signaling pathways. Future research should investigate whether autophagy is affected by the degree and duration of bone defects at high altitudes.

### Epigenetics

Populations living at high altitudes for generations all have a genetic basis for adaptation to high-altitude environments. However, genetics alone cannot fully explain the mechanism of impaired bone defect repair at high altitudes. An increasing number of studies have confirmed that epigenetic alterations integrate genetic and environmental stimuli to participate in hypoxia adaptation of tissues and cells, thereby affecting bone defect repair by regulating related downstream genes. Some of the most prominent epigenetic mechanisms include DNA methylation, histone modifications, and non-coding RNAs.

The most intensively studied epigenetic mechanism to date is DNA methylation. DNA sequencing in high-altitude and plain populations reveals that genes with differential methylation are mainly involved in HIF-related signaling pathways (98, 99). The methylation levels of HIF-1α and HIF-2α promoter regions are significantly lower in plateau animals (99, 100). HIF-dependent hypoxia response element (HRE)-related genes are sensitive to methylation, and a large number of CpG dinucleotides are present at the binding site of the HRE sequence of HIF (101). When methylation changes occur at the above sites, binding to various transcription factors is inhibited. Studies have shown that chronic hypoxia induces CpG for VHL promoter methylation, reduces VHL expression, and increases EPO production by elevating HIF-2α expression in the bone marrow, leading to erythrocytosis (102). Meanwhile, HIF-1α cannot be degraded by ubiquitination and further accumulates in cells (103). Evidence suggests that DNA hypomethylation in high-altitude environments favors the expression of *HIF* genes, in turn triggering the hypoxia adaptation response of the tissue and cells by regulating the activity of downstream genes.

It is known that high-altitude hypoxia also leads to changes in histone modifications. The modification sites are generally located on four common histones, including H2A, H2B, H3, and H4—with H3 and H4 being the most common. Studies have shown that high-altitude environments increase the levels of H3K14ac, H4R3me2, H3K4me2, H3K4me3, H3K79me2, H3K9/27me2, H3K9me2, H3K27me3, and H3K4mel in mice (104). In addition, the Jumonji domain (JMJD) protein family comprises enzymes catalyzing the demethylation of arginine and lysine residues in histones. Both JMJD1A and JMJD2B retain their activities, bind to specific recognition sites of HIF-1α, and induce its expression under high-altitude hypoxia (105). The chemical modification of these histones ultimately alters the expression of the genes by changing the affinities of the promoter regions of genes associated with hypoxia response.

Non-coding RNAs are expressed differently in plateau and plain environments. Examining the expression profiles of miRNAs between these environments revealed a total of 26 differentially expressed miRNAs (106). The hypoxic environment upregulates the expression of miRNA-21 (107) and miR-486 (108), while increasing osteogenic differentiation of bone MSCs (BMSCs) through the PI3K/Akt pathway. Studies have shown that lncRNAs play an important role in the direct or indirect regulation of HIF-1α expression and related pathways, and interrupt angiogenesis and bone formation in hypoxia by negatively regulating HIF-1α at the mRNA level. The lncRNA *KCNQ1OT1* exerts the primary effects in delayed fracture healing, inducing cell proliferation and inhibiting cell apoptosis by activating the Wnt/β-catenin signaling pathway (109). In addition, hypoxia promotes the production of miR-126 production in exosomes to enhance bone defect healing *via* the SPRED1/Ras/ERK signaling pathway (110).

It remains controversial whether epigenetic factors are beneficial to bone defect repair at high altitudes. Most studies investigating the impact of epigenetic factors started from the aspect of hypoxia in the high-altitude environment. However, it is important to identify other key factors involved in the healing of bone defects to determine how they are regulated under a high-altitude environment. Moreover, the histone modification process is extremely complex and has not been comprehensively studied. For example, the histone protease LSD1 is a key factor regulating endochondral ossification during bone regeneration (111). Yet, how it changes under high-altitude environments has not been elucidated, which deserves further attention.

## Implications For Treatment

### Oxygen Therapy

Oxygen therapy is the primary treatment for acute and chronic altitude sickness. It primarily serves to improve PaO_2_ and arterial oxygen saturation, increase the content of arterial oxygen, and correct various hypoxia levels caused by the high altitude. Furthermore, oxygen administration can be divided into systemic and local types.

The most common systemically administered oxygen therapy is HBOT (112). Hyperbaric oxygen can reduce the cardiac workload, improve cardiac function, block the vicious cycle of hypoxia leading to excessive erythrocyte proliferation, reduce the respiratory rate, and correct the acid-base balance. Hyperbaric oxygen can play a role in promoting the repair of both fresh and old bone defects. It helps reduce tissue edema, restore venous return, improve microcirculation (113, 114), and stimulate angiogenesis (115), Furthermore, it also increases PaO_2_ in the fractured area (especially in the callus and medullary cavity), enhances the activities of osteoclasts and osteoblasts, and accelerates bone callus formation (116). In addition, hyperbaric oxygen also enhances the anti-infective ability of local tissues, especially against anaerobic bacteria (117). Clinically, hyperbaric oxygen chambers have been widely used in patients with bone defects to shorten the recovery time. In response to peri-implant tissue stimulation by titanium (Ti) particle exposure, ROS production (118), pro-inflammatory cytokines, infiltration of inflammatory response cells, and activation of the osteoclast activity (119), HBOT can be used in combination with bone grafts (120). Lastly, HBOT also improves the implantation of osseointegration (121).

Topical oxygen therapy (TOT) has been used in diabetic skin ulcers, post-operative infections, and gangrenous lesions. The most common method uses the micro-oxygen wound therapy instrument, which delivers pure oxygen to the wound for 24 h *via* an oxygen administration tube yet is not readily applied in bone defect repair. Studies have also found that local oxygen administration improves post-operative local PaO_2_ and oxygen saturation at sternal defects and reduces the risk of infection (122). In addition, reports have adopted innovative local oxygen delivery methods. For instance, perfluoro-octane-loaded hollow particles can be used as a local oxygen source, increasing cell viability, and maintaining the osteogenic differentiation potential of human periosteum-derived cells under hypoxic conditions (123, 124). However, only local oxygen-releasing dressings, including OxygeneSys, Oxyzyme, and Oxyband, have been marketed to date, and oxygen released from these products can only act on the wound surface and cannot improve PaO_2_ to address deep bone defects.

In general, HBOT has been widely used in clinical practice with a definite curative effect, but measures should be taken according to local conditions. Specifically, the treatment pressure should not be too high, and the speeds of pressure increase and decrease rates should be reduced, with the times of pressure increase and decrease extended appropriately. In addition, the high-pressure oxygen chamber has various shortcomings, including inconvenient mobility, which cannot meet the criteria of emergency treatment to combat altitude sickness. Thus, scientists have also developed a vehicle-mounted mobile hyperbaric oxygen chamber. However, the effect of local oxygen therapy in the treatment of bone defects remains unclear and deserves further investigation.

### Systemic Administration

Some drugs can promote the healing of bone defects by improving the physiological ability to adapt to hypoxic environments, reducing ROS, and improving ion permeability of the cell membrane.

As far as traditional Chinese medicine (TCM) is concerned, the anti-altitude sickness drug rhodiola has the obvious effect of inhibiting bone resorption. Studies have found that rhodiola reduces ROS production, significantly decreases the expression and activity of MMPs, and upregulates the TIMP protein (125). Salidroside, the major bioactive compound of rhodiola, has various pharmacological effects and acts through HIF-1α-VEGF (126) and BMP (127) pathways, simultaneously promoting angiogenesis and osteogenesis, thereby accelerating bone defect healing. Rosavin, a rhodiola component, can block the NF-κB and MAPK pathways, inhibit RANKL-induced osteoclast formation *in vitro* and *in vivo*, decrease the expression of genes related to osteoclast differentiation, and promote osteogenesis in BMSCs (128). In addition, Jiuerjiegusan and Jieguling capsules are used in the treatment of high-altitude traumatic fractures, also significantly accelerating fracture healing.

Supplementation of antioxidants can reduce the negative effects of oxidative stress on bone defect repair by reducing ROS amounts (129). Common oral exogenous antioxidants include vitamin C, vitamin E, and trace elements. Polyphenols in fruits and vegetables also act as natural antioxidants. The effects of oral antioxidants are currently controversial. Studies have found that antioxidant cocktail therapy has no effects on bone resorption or formation (130). Epidemiologic studies showed that although systemic administration of vitamin C improves the soft tissue healing of tooth extraction wounds (131, 132), there is no significant difference in the percentage of X-ray density of new bone formation (133). The main polyphenolic catechins in green tea increase the survival, proliferation, increasesdifferentiation, and mineralization of osteoblasts by promoting osteogenic differentiation of MSCs (134). Conjugated linoleic acid (CLA) is an important component of the Tibetan diet, with a strong antioxidant effect. CLA the quality and mechanical strength of bone callus fracture healing in rats (135), and significantly reduces alveolar bone loss in rats with periodontitis and diabetes (136).

Calcium ion antagonists, which dilate blood vessels and relax bronchial smooth muscles, are commonly used to treat high-altitude reactions, improving the anti-hypoxia ability to various degrees. Ronacaleret, a novel calcium-sensing receptor antagonist, stimulates the release of parathyroid hormone (PTH) and increases the expression of bone formation markers. A phase I and II clinical study found that it acts as a potent oral anabolic agent to promote bone fracture healing (137). However, it has not been reported whether other calcium antagonists can be used for the treatment of high-altitude-related bone defects by systemic administration.

Most of the above reports are basic research, and large-scale, placebo-controlled, long-term randomized trials with optimal timing of protocol interventions are still needed to determine the efficacy of drugs on bone defect repair. In addition, non-targeted mitochondrial antioxidants cannot accumulate in large quantities in key steps of mitochondrial ROS production, and may eventually interfere with subsequent physiological signal transduction (138). Therefore, the safety of these proof–of–concept drugs remains to be confirmed in further experiments. Under the guidance of the TCM theory, TCM has its advantages in fundamentally preventing and treating altitude sickness. Its combined application with Western medicine is expected to become the future therapeutic standard for treating plateau bone defects.

### Local Drug Delivery

Drugs directly acting on bone defects are administered by local drug delivery. With the development of biomaterials and bone tissue engineering technology, local drugs are sometimes combined with biomaterials to enhance angiogenesis and osteogenesis abilities, which effectively repair bone defects and promote bone regeneration to a certain extent.

Bioactive materials have been widely reported in the treatment of bone defects, but whether they have the same efficacy in high-altitude environments remains to be confirmed. In view of the potential mechanism of poor bone defect repair at high altitudes, existing studies can serve as a certain reference. Amorphous silicon nitride (Si(On)_x_) can be used as nano-coating for titanium plates, which have a strong attachment to the surface of scaffolds and induces the sustainable release of Si (+4-valent) for a prolonged time (139), enhancing the expression of superoxide dismutase. Thus, its therapeutic use is also expected to promote bone tissue repair and angiogenesis by reducing the influence of ROS in the high-altitude environment (140). Bioactive borosilicate glass (BG) scaffolds and tricalcium phosphate (TCP) are commonly used materials in bone repair. DMOG, a small molecule angiogenic drug that can adjust the stability of HIF-1α (141), can be added to BG and TCP to promote new bone formation and neovascularization in bone defects (142).

HIF is a key factor in response to hypoxic stress in high-altitude environments; direct application of HIF or the use of HIF-related drugs may promote bone defect repair at high altitudes. For instance, DBBM-C (deproteinized bovine bone +10% collagen) combined with HIF-1α promotes the formation of new bone (143). HIF-1α mediates DNA delivery *via* the protein transduction domain (PTD), and local administration of HIF-1α *via* PTD promotes bone growth (142). Under severe hypoxia, mechanical growth factor E inhibits the expression of HIF-1α and its transfer to the nucleus, thus regulating the proliferation and osteogenic differentiation of BMSCs (144). In addition, local application of HIF-related drugs may also be helpful for cartilage repair. Studies have shown that HIF-1α combined with collagen scaffold can repair osteochondral defects of the condyle of the temporomandibular joint in rabbits (145). Local injection of icariin inhibits the NF-κB/HIF-2α signaling pathway, thereby enhancing chondrocyte viability (146).

As discussed earlier, autophagy plays an important role in poor bone defect repair at high altitudes. Therefore, applying drugs regulating autophagy may be a potential therapeutic approach. Metformin, which is commonly used to combat hyperglycemia, has also been shown to affect bone regeneration. Metformin increases autophagy in BMSCs under hypoxic conditions and upregulates the osteogenic markers Runx2, osteocalcin, and alkaline phosphatase to significantly accelerate the formation of new bone (147, 148). Simvastatin, a serum cholesterol-lowering drug, has been shown to promote bone regeneration. Topical administration of simvastatin enhances autophagy and reduces the activity of osteoclasts (149), as well as induces homing of endothelial progenitor cells and promotes angiogenesis (150). It is as effective in repairing long tubular and flat bone defects as autografts (151). Other studies have found that under hypoxic conditions, resveratrol and angiopoietin 2 improve the survival and differentiation of BMSCs through autophagy (152, 153). Furthermore, intraperitoneal administration of the autophagy inducer rapamycin in fractured rats improves the autophagy level, increases bone callus formation, and accelerates fracture healing (154). Nevertheless, rapamycin has many adverse reactions. Therefore, identifying autophagy modulators with reduced toxicity and good efficacy is a direction of future research.

Local drug therapy can act directly on the bone defect site to accelerate fracture healing and enhance bone graft stability. However, the condition of bone defects faced by clinicians in high-altitude environments is complex and often occurs in combination with hypothermia, hypoxemia, and infection. Therefore, it is possible to combine drugs that promote osteogenesis with those conferring anti-infection and rehydration to establish a suitable rescue treatment approach for bone defects in high-altitude areas.

### Stem Cells

The regeneration ability of bone tissue cells is compromised in high-altitude environments. Fortunately, stem cell therapy by itself or in combination with drugs, surgery, biomaterials, etc., can play a role in accelerating wound repair by improving tissue differentiation ability.

In high altitude environment, EPO was increased. Erythropoietin receptor (EPOR) was expressed in bone marrow stromal cells (BMSC), and it was verified that increased EPO results in reduced bone by regulating BMSCs (155). Although the local transplantation of BMSCs accelerated wound repair of high-altitude femoral defects, the healing rate of the high-altitude group remained lower than that of the plain group (156). Therefore, improving the survival rate and osteogenic ability of transplanted BMSCs in hypoxic regions is crucial. Studies have shown that BMSC transplantation combined with FG4592 administration can further accelerate fracture healing by increasing the proliferation and migration of BMSCs (157). BMSCs are reprogrammed into induced pluripotent stem cells (iPSCs), called iPSC-MSCs, whose morphology, immunophenotype, *in vitro* differentiation potential, and DNA methylation pattern are similar to those of BMSCs. Nevertheless, iPSC-MSCs have a higher proliferative capacity and promote bone repair and angiogenesis more pronouncedly (158). A 3D cell oxygen permeation culture device “Oxy Chip” was developed to generate and supply oxygen to cell spheroids to prevent hypoxia (159). It could promote osteoblastic differentiation of MSCs.

Adipose-derived stem cells (ADSCs) could differentiate into chondroblasts after 2 days of *in vitro* culture without the addition of growth factors at low oxygen partial pressure (<1% PO_2_) (160). However, for osteogenic differentiation to be achieved, different growth factors or biomaterials should be combined to provide an osteogenic induction environment for ADSCs. The combination of ADSCs and autologous platelet-rich plasma (PRP) was not statistically significant compared with autologous bone grafts (161). This may be because the growth factor mixture in PRP has a short half-life *in vivo*, which cannot guarantee osteogenic induction of ADSCs during the whole process of bone repair. Nonetheless, tissue engineering scaffolds have advantages in providing a continuous environment for osteogenic induction. Some scholars have developed epigallocatechin gallate (EGCG)-coated synthetic fibers encapsulating ADSCs to fabricate stem cell spheroids for bone tissue regeneration. These EGCG fibers effectively delivered osteo-inductive and ROS scavenging signals to ADSCs in spheroids, upregulating the osteogenic markers RUNX2 and OPN (162). In addition, a novel 3D BG scaffold (BG-XLS/GelMA-DFO) combined with ADSCs could also promote bone regeneration under simulated hypoxia conditions (163).

The cell viability and survival time of ordinary 2D-cultured MSCs after transplantation is not ideal. It is the current trend to aggregate cells into 3D spheres or to bind, extend, and grow them on porous 3D scaffolds. Since blood supply plays a crucial role in bone defect repair, whether the 3D culture of stem cells can be vascularized has become an important hotspot for research. Organoids are stem cell-derived 3D culture systems. It is now possible to re-create the architecture and physiology of human organs in remarkable detail. A preliminary breakthrough has shown that organoids can form a vascular network through the Organ Bud technology (164, 165), which is expected to become a future research direction for developing treatment tools for bone defects at high altitudes by stem cell transplantation.

### Gene Modifications

The rapid development of modern molecular biology theory and technology has given rise to innovative solutions to address bone defect repair. For instance, using gene therapy, a target gene combined with a carrier can be injected directly into the target tissue. Alternatively, gene-modified stem cells can be used to promote new bone formation and repair bone defects. The most common methods of gene modifications are viral and non-viral vectors, including retroviruses, lentiviruses, adenoviruses, liposomes, and cationic polymers.

Osteogenesis-related genes can be introduced to target cells by vectors. This enables achieve long-term stable expression of the genes of interest within the bone defect, which could promote bone defect repair. Studies have shown that adenovirus transduction of human *BMP2* promotes osteogenic differentiation of adipose tissue fragments (166). Introducing the *BMP2* gene into BMSCs can induce bone differentiation and accelerate the healing process (167–169). Furthermore, using gene modification, the *OPG* gene was introduced into BMSCs seeded on a hydroxyapatite (HA) scaffold to form a novel OPG-BMSC-HA complex, which could promote the osteogenic effect of BMSCs and facilitate bone defect reconstruction therapy (170). *Runx2* gene-modified MSC-derived 3D spheroids have also been used to effectively promote bone regeneration (171). VEGF transfected with recombinant adenovirus vector preserves the oxygen sensitivity of HIF-1/HRE and promotes vascularization (172).

As small non-coding RNAs that regulate gene expression, miRNAs have become new targets for poor bone healing. Studies have shown that ADSCs transfected with miR-26a (173), as well as BMSCs transfected with miR-218 (174) and miR-29b-3p (175), can improve bone regeneration capacity. In addition, miR-378 can stimulate both osteogenesis and angiogenesis (176). Therefore, it can be used as a reference target for the treatment of bone defects at high altitudes (177). Regarding the mechanism underlying the effect of high altitude on bone repair, the hypoxic environment can upregulate miR-21 (107) and miR-486 (108). Therefore, these two miRNAs are also potential therapeutic targets. It was found that miR-21 activates the PI3K/Akt signaling pathway (178, 179) and significantly increases the volume of new bone formation and mineralization at the bone defect site. miR-486-3p targeting catenin β-interacting protein 1 can activate the Wnt/β-catenin signaling pathway and promote the bone formation of BMSCs (180).

Exosomes exhibit biological characteristics similar to those of the parent cells and can be used as carriers to deliver genes to cells (181). In addition, direct application of exosomes can also reduce the risk of immunogenicity, avoiding the ethical and technical issues linked to cell therapy (182). BMSC-derived exosomes carrying miR-335 promote fracture recovery through activation of the Wnt/β-catenin signaling pathway (183) and enhance bone regeneration by inhibiting hypoxia-induced osteocyte apoptosis (184)—thus, significantly preventing bone loss and increasing the blood vessel volume of the femoral head (185). Ther exosomal release is induced upon hypoxia (186) and this approach may be used in the treatment of bone defects in high-altitude-related hypoxic environments. In addition, umbilical cord MSC-derived exosomes increase the expression of VEGF and HIF-1α, which may accelerate fracture healing by promoting angiogenesis (181). The combination of exosomes with biomaterials is also one of the potential therapeutic strategies for repairing cartilage (187) and bone defects (188). Lastly, a novel “NANOBIOME” approach based on the biobanking of exosomes secreted by MSCs has shown promise as an innovative “cell-free” regenerative medicine (182).

Genetic regulation in organisms is a highly sophisticated dynamic process. The safety of gene-modified therapies in humans still needs consideration as their improper use may lead to cell dysfunction and other side effects. For example, miRNAs can be involved in multi-target regulation and may cause adverse reactions in tissues other than the bone when applied *in vivo*. In addition, human manipulation of epigenetic regulation requires higher legal requirements. Therefore, convenient, rapid, efficient, and safe epigenetic modification methods are required to achieve accurate treatment of bone defects at high altitudes.

## Conclusions and Recommendations For Future Research

In most *in vitro* experiments, researchers applied oxygen concentrations of 1–5% as hypoxic conditions and used oxygen concentrations of ~21% at standard atmospheric pressure for comparison. However, the latter is indeed much higher than the oxygen concentration in the physiological state of the tissue microenvironment, whereas the former is close to normal physiological conditions in humans [e.g., the oxygen concentration in the bone marrow is 4–7% (189)]. Furthermore, studies examining high-altitude environments are often limited to reduced oxygen content which can be misleading. First, actual oxygen concentration at the plateau is not low as all oxygen levels are ~21% between sea level to an altitude of 100,000 m. Altitude sickness is actually caused by a drop in PaO_2_. At this elevation, hypoxia occurs when PaO_2_ in the air inhaled by the human body falls below 16 kPa (2,500–3,000 m). Since the key molecule in oxygen sensing and adaptation is HIF-1, it may be a more accurate method to simulate the effect of high altitude on cells by regulating the expression of intracellular HIF-1. For example, prolyl hydroxylase inhibitors can be used to effectively increase HIF-1α protein stability (190).

At present, there is no clear optimal treatment plan for bone defects at high altitudes. The existing clinical work in high-altitude areas is mainly based on systemic oxygen therapy and bone transplantation. This is because many treatment methods involving signaling pathways and gene modifications are still in the basic research stage. Therefore, drugs targeting mitochondrial dysfunction and novel bone graft materials with oxygen-carrying capacity are urgently needed. Elabel (ELA), a peptide hormone, has been shown to promote the growth, survival, and pluripotency of human embryonic stem cells (191, 192). However, to date, ELA has only been studied for the treatment of cardiovascular diseases (193, 194), and its therapeutic effect on bone defects at high altitudes requires investigation.

As biomaterial and bone tissue engineering technology is advancing, developing biomaterials for plateau environments could be a future research hotspot. A new type of bone graft with an oxygen-carrying function can provide both local oxygen supply and bone substitute. Wang et al. (195) developed a novel oxygen sustained-release biomaterial composed of CaO_2_/gelatin microspheres and a 3D printed polycaprolactone/nano-HA composite porous scaffold. Their results showed that CaO_2_/gelatin microspheres continuously released oxygen for 19 days, improving the survival rate of transplanted BMSCs in the rabbit model by reducing local apoptosis. However, further experiments evaluating high-altitude animal models are needed to confirm the repair effect of this material on bone defects.

With the discovery of mitochondrial dysfunction, autophagy, signaling pathways, and epigenetic mechanisms of high-altitude bone defects, the development of better, targeted, personalized, and precise bone defect repair methods adjustable according to the high-altitude adaptation of patients should be expected.

## Author Contributions

MR and ZL conceptualized this manuscript. YW and PC retrieved and interpreted the information. PC and YL wrote the manuscript. WL revised the manuscript. All authors contributed to the article and approved the submitted version.

## Funding

This study was funded by the Natural Science Foundation of Tibet Autonomous Region (Grant No. XZ2017ZR-ZY37); Guangdong Province Science and Technology Innovation Strategy Special Fund Project (Grant No. 2018KJY2014); Scientific Research Project of Southern Medical University Stomatological Hospital (Grant No. PY2018014), and the Medical Scientific Research Foundation of Guangdong Province of China (Grant No. A2020435).

## Conflict of Interest

The authors declare that the research was conducted in the absence of any commercial or financial relationships that could be construed as a potential conflict of interest.

## Publisher's Note

All claims expressed in this article are solely those of the authors and do not necessarily represent those of their affiliated organizations, or those of the publisher, the editors and the reviewers. Any product that may be evaluated in this article, or claim that may be made by its manufacturer, is not guaranteed or endorsed by the publisher.
